# *IL10* rs1800872 Is Associated with Non-Steroidal Anti-Inflammatory Drugs Exacerbated Respiratory Disease in Mexican-Mestizo Patients

**DOI:** 10.3390/biom10010104

**Published:** 2020-01-07

**Authors:** Gandhi Fernando Pavón-Romero, Gloria Pérez-Rubio, Fernando Ramírez-Jiménez, Enrique Ambrocio-Ortiz, Cristian Rubén Merino-Camacho, Ramcés Falfán-Valencia, Luis M. Teran

**Affiliations:** 1 Department of Immunogenetics and Allergy, Instituto Nacional de Enfermedades Respiratorias Ismael Cosío Villegas, Calzada de Tlalpan 4502, Sección XVI, Tlalpan, Ciudad de México 14080, Mexico; gandhifernando@hotmail.com (G.F.P.-R.); dr_frj@yahoo.com.mx (F.R.-J.); crismerino44@gmail.com (C.R.M.-C.); 2 HLA Laboratory, Instituto Nacional de Enfermedades Respiratorias Ismael Cosío Villegas, Calzada de Tlalpan 4502, Col. Sección XVI, Tlalpan, Ciudad de México 14080, Mexico; glofos@yahoo.com.mx (G.P.-R.); e_ambrocio_iner@hotmail.com (E.A.-O.)

**Keywords:** *IL10*, SNP, N-ERD, genetic association

## Abstract

Non-steroidal anti-inflammatory drugs (NSAID) exacerbated respiratory disease (N-ERD) is a disease integrated by asthma, nasal polyps, and hypersensitivity to non-steroidal anti-inflammatory drugs (NSAID). Genetic association studies have explored single nucleotide polymorphisms (SNPs) in genes involved in theoretical pathophysiological mechanisms, but most of these lack replication of findings in second populations. Our objective was to evaluate the association of SNPs in candidate genomic regions described in Asian and European subjects with N-ERD in Mexican-mestizo patients. We designed a replicative study in two stages. We included 381 SNPs selected by fine mapping of associated genes in a microarray, which were tested in three groups: N-ERD (N), asthma (A), and control group (CG); by means of GoldenGate array, positive results by genetic models were validated in the second stage in another population through qPCR with the same methodology. In the allelic model, we identified 11 SNPs in N vs. CG comparison, and five in N vs. A and A vs. CG, respectively. By genetics models, all SNPs in *PPARG*, rs13239058 in *TBXAS1*, and rs1554286 and rs1800872 in *IL10* were associated in both models. In the second stage, only rs1800872CC showed an association in the dominant model comparing N vs. GC, *p* = 0.004, OR = 0.44. In conclusion, rs1800872 in *IL10* was the only associated with N-ERD in Mexican-mestizo patients.

## 1. Introduction

Non-steroidal anti-inflammatory drugs (NSAID) exacerbated respiratory disease (N-ERD) is an etiology integrated by asthma, chronic rhinosinusitis with nasal polyps, and intolerance to NSAID [1]. It is estimated that it is present in 7% of patients with asthma and increases twice in cases of severe asthma [2].

The physiopathology is unclear yet, principally is oriented in a didactic way to the mechanism of action of NSAID, blockage of the cyclooxygenase pathway deriving the metabolic substrate from arachidonic acid (AA) to the lipoxygenase pathway, with subsequent overproduction of leukotrienes (LTC4, LTD4, and LTE4) that induce typical symptoms of N-ERD such as nasal constipation and bronchospasm before intake of NSAID [3]. Recently, other mechanisms have been integrated, such as epithelial damage mediated by thymic stromal lymphopoietin with activation of the innate type 2 immune system [4,5] and the role of enterotoxins of *Staphylococcus aureus* in airway inflammation [6].

From 1997 several genetic studies on aspirin hypersensitivity have been performed to discover the genetic predisposition to aspirin hypersensitivity [7], prevailing two kinds of studies, those that evaluated single nucleotide polymorphisms (SNP) in candidate genes with a putative or evident mechanism in N-ERD as *ALOX5* [8], *LTC4S* [7], *CYSLTR1* [9], *CYSLTR2* [10], *COX-2* [11], among others, and the genome-wide association studies that analyze the association of many genetic variants looking for alternative hypotheses in genes other than candidate functional variants such as HLA-DPB1 [12]. Independent of study type, both have strengthened the understanding of pathophysiological mechanisms and identify disease phenotypes of N-ERD; recently epigenetic changes have been explored [13].

Our group replicated 53 candidate genomic regions spanning over 19 chromosomes reported in N-ERD, through Tag SNP strategy (311SNP), finding a new SNP (rs573790) associated to risk in the *MS4A2* gene with N-ERD in Mexican-Mestizo patients [14]. In this report, our aim is to describe the association of SNPs to the protective effect.

## 2. Materials and Methods

### 2.1. Study Design and Study Groups

**Figure 1:**
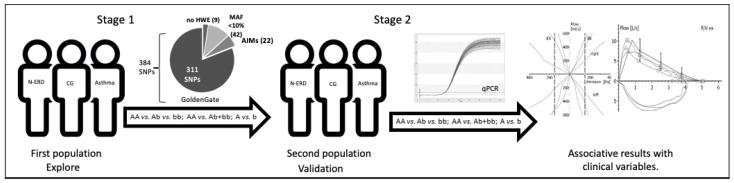
N-ERD non-steroidal anti-inflammatory drugs exacerbated respiratory disease. CG: control group. No-HWE: no Hardy–Weinberg equilibrium. MAF: minor allele frequency. AIMs: ancestry informative markers, qPCR: quantitative polymerase chain reaction.

We developed a replicative study in two stages. In the first, we evaluated SNPs selected by fine-mapping regions associated positively with N-ERD using the GoldenGate array (Illumina, Inc., San Diego, CA, USA), and only the associated results (*p* < 0.05, OR < 1) were considered for validation prior to an internal group consensus. In the second stage with another population of subjects by allelic discrimination using qPCR and its association with clinical variables (Figure 1).

We enrolled for the two stages Mexican-mestizo subjects, defined as being born in Mexico and with Mexican ancestry (at least two previous generations), N-ERD was defined as the presence of symptoms of chronic rhinosinusitis with nasal polyps (presence or antecedent of white or pink grapelike masses in the nose), plus the condition of intolerance to NSAID defined as positive nasal challenge with Lysin-aspirin (decreased of total nasal flow (TNF) >40% according to basal values, before 100 mg of Lysin-aspirin) or antecedent of two severe reactions i.e., asthmatic crisis after the intake any type of COX-1/NSAID, documented in medical records, this kind of criteria were used for enrolling the patients in both stages and asthma was established as persistent symptoms: cough, chest tightness, wheezing, etc., plus an increase of ≥12% plus 200 mL of forced expiratory volume in the first second (FEV1), before 400 µg of Salbutamol in post-bronchodilator spirometry (MasterScreen, Jaegger. Bonn, Germany), this is the only characteristic that the N-ERD group shares with the asthma group. If the subjects had no clinical symptoms or positive tests, they were integrated into the healthy control group (CG). All subjects were enrolled in the outpatient clinic at the Mexican Instituto Nacional de Enfermedades Respiratorias Ismael Cosio Villegas (INER).

Independently of the group, we evaluated allergy sensitization with a skin prick test, (Alk-abello, Round Rock, TX, USA), the measure of total IgE levels (Architect i2000, Abbott, Germany), and eosinophils count by hematic cytometry (Beckman Coulter LH750, Brea, CA, USA).

All participants were previously invited to participate in the study; they signed an informed consent document and were provided with a privacy statement describing the protection of personal data. The Institutional Ethics and Research Committees reviewed and approved the study with number B14-12. All names and respective samples of subjects of the study were codified with alphanumeric-ID, making their identification impossible and safeguarding their personal information, according to Mexican laws (Ley General de Salud in human research—articles 16 and 21—and INER-04-08-88).

### 2.2. DNA Isolation

We obtained from study subjects 8 mL of peripheral blood by venipuncture collected in a tube with EDTA as the anticoagulant, and subsequent DNA extraction was performed using a BDtract DNA Isolation Kit (Maxim Biotech, San Francisco, CA, USA). The DNA was quantified by ultraviolet absorption at a 260 nm wavelength using a Nanodrop instrument (Thermo Scientific, Wilmington, DE, USA). All samples were adjusted to 50 µg/µL for subsequent genotyping.

### 2.3. SNP Selection

For SNP selection for microarray integration, we did a search of SNPs associated to AERD (Aspirin-Exacerbated Respiratory Disease, currently known by consensus from 2018 as N-ERD) between 1997 and 2014 in the US National Library of Medicine with the keywords SNP and AERD (aspirin exacerbated respiratory disease), aspirin-tolerant asthmatics (ATA) hypersensitivity and SNP, asthma intolerance to aspirin and genetics. The array included 384 SNPs from 53 candidate genomic regions spanning over 19 chromosomes, of which 63 SNPs were associated with N-ERD, 299 were tag SNPs, and 22 SNPs were ancestry informative markers (AIMs). The selection criteria of the SNPs were based on the minor allele frequency (MAF) >10% in the Mexican mestizo population (data obtained from the Mexican genome diversity project, MGDP) and with Hardy–Weinberg equilibrium *p* > 0.05.

### 2.4. Genotyping and Quality Control

Genotyping was conducted using the protocol designed by Illumina for the GoldenGate platform (Illumina, Inc., San Diego, CA, USA) using a Tecan robotic automatic liquid dispenser (Tecan, Trading AG, Männedorf Switzerland), which operates under the Illumina protocol. The microarrays were read on the BeadArray Reader scanner (Illumina, Inc., San Diego, CA, USA). Genotype acquisition and generation of documentation (ped and .map files) were conducted using the GenomeStudio2011 v1.0 software (Illumina, Inc., San Diego, CA, USA). Subjects who did not comply with the call rate criteria (>95%) were excluded.

### 2.5. TaqMan Allelic Discrimination

Allelic discrimination of SNPs was performed by real-time PCR (RT-PCR) on a 7300 Real-Time PCR System (Applied Biosystems, Foster City, CA, USA) using TaqMan commercial probes (Applied Biosystems, Foster City, CA, USA) for each of the polymorphisms mentioned above and followed the cycling program: pre-read 50 °C, 1 min; absolute quantitation: 50 °C, 2 min, one cycle; 95 °C, 10 min, one cycle; 95 °C, 15 s, 60 °C 1 min, 40 cycles; post-read 50 °C, 1 min. The results were assessed considering the allelic discrimination and absolute quantitation in all samples; additionally, we included four contamination controls per plate (non-template controls). The interpretation was performed with Sequence Detection Software (v. 1.4). The fluorescence signal detectors used were VIC which was assigned to the B allele and FAM assigned to the A allele for both SNPs.

### 2.6. Statistical Analysis

Clinical quantitative variables were entered and analyzed using SPSS for Windows, version 21 (SPSS software, IBM, Chicago, IL, USA). Descriptive statistics were used, and comparisons between qualitative data were made using chi-square tests to gauge significance. A p value of less than 0.05 was considered statistically significant. The frequency analysis (clinical and genetics) was performed with Epi-info software v.7.0. In the first stage only, the genetic analysis of minor allele frequency was performed with PLINK software [15], subsequently, reanalyzing according to genetic models, the codominant and dominant models were performed using the Epidat version 3.1 software and Epi-info v7.2 software. In case that one gene had more than one SNP, we undertook a haplotype analysis with Haploview 4.2 software. In the second stage, we only applied analyses to allele and genetic models. The fixation index (Fst) was determined using EIGENSOFT v4.2 software [16]. We performed a bivariate logistic regression with the main genetic findings, in all analyses, we considered significance at a *p* < 0.05.

## 3. Results

### 3.1. First-Stage Analysis

#### 3.1.1. Demographic and Clinical Characteristics

We enrolled in the first phase 120 patients with N-ERD, 180 with asthma, and 180 control subjects, the female gender prevailed in the three groups with a statistical difference between asthma vs. CG (*p* = 0.04); regarding the age of the patients, those in N-ERD and asthma groups were around the fourth decade of life and were older than CG (*p* < 0.001), the eosinophil counts were higher in N-ERD compared with asthma and control groups (*p* < 0.001), but serum IgE levels were higher in asthma patients vs. N-ERD and CG (*p* < 0.001 by each comparison). No difference in allergic sensitivity by skin test was reported in the three groups (*p* > 0.05), the best the lung function was in CG in comparison with patients (*p* = 0.002) and did not show statistical significance between N-ERD vs. asthma groups. Regarding the nasal challenge with lysine aspirin only the positivity criteria was met in the NERD group (*p* < 0.001) (see Table 1).

#### 3.1.2. Ancestry

All subjects in the study had a similar proportion of genetic ancestry according to the two principal population groups that integrate the Mexican-mestizo population (AME—Amerindian and CEU—Caucasian) the Amerindian component prevailing slightly; by group, we describe the following frequencies: AERD had 52% of AME and 48% of CEU, asthma had 56% AME and 44% CEU, and CG had 58% AME and 41% CEU. The Fst test did not identify differences among the three groups, but there was a difference when the groups were compared with CEU and AME ancestry markers (*p* = 0.005) (see Table 2).

#### 3.1.3. Allelic Model by GoldenGate

In the comparison N-ERD vs. CG, nine SNPs in five genes (*PPARG*, *IL10*, *TBXAS1*, *FCER1G*, and *FANCC*) showed an association, the rs2960421 in *PPARG p* = 0.002, OR = 0.48, CI95% (0.30–0.77) and rs155422 of *IL10 p* = 0.003, OR = 0.60, CI95% (0.42–0.84) were the most strongly associated SNPs. In N-ERD vs. asthma comparison, we detected five SNPs in four genes (*PPARG, IL10, PTGER2*, and *OBSCN*). *IL10* contributed with two polymorphisms in the first places, rs155422 and rs18000872 *p* = 0.006, OR = 0.62, CI95% (0.44–0.87) and *p* = 0.02, OR = 0.68, CI95% (0.49–0.96), respectively. In the last comparison asthma vs. control group we detected seven SNPs in four genes (*TBXAS1*, *FANCC*, *CYSLTR2*, and *PTGER3*). rs2072190 and rs226997 in *TBXAS1* were the SNPs with the greatest association *p* = 0.001, OR = 0.61, CI95% (0.45–0.83) and *p* = 0.006, OR = 0.66, CI95% (0.49–0.89) (see Table 3).

#### 3.1.4. Haplotype Analysis 

**Figure 2:**
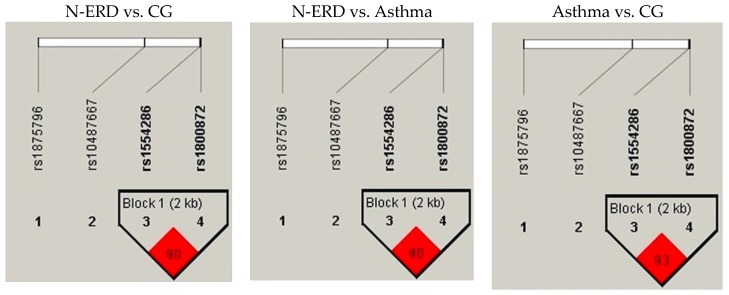
Haplotype analysis of *IL10* (rs155286 and rs1800872). N-ERD: non-steroidal anti-inflammatory drugs exacerbated respiratory disease. CG: control group.

The SNPs of *IL10* rs1554286 and rs1800872 had a high linkage disequilibrium in the tree comparisons, N-ERD vs. CG and N-ERD vs. asthma had r^2^ = 0.90, and asthma vs. CG had r^2^ = 0.93 (see Figure 2). Regarding global haplotype frequency, the segregation of alleles CC was predominant (57.8%), then TA (39.7%) and CA (2.5%).

The haplotype CC conferred a statistical association in the comparison N-ERD vs. CG *p* = 0.01, OR = 0.65, (CI95% 0.47–0.92), and N-ERD vs. asthma, *p* = 0.04 OR = 0.69, (CI95% 0.49–0.97); we detected a similar magnitude with TA alleles in the aforementioned comparisons (Table S1). The two SNPs in *IL10* are in Hardy–Weinberg equilibrium, rs1554286, *p* = 0.78 and rs1800872, *p* = 0.80. We did not identify linkage disequilibrium with the SNPs of *PPARG* and *TBXAS1* (Figure S1).

#### 3.1.5. Genetic Models Analysis in the First Stage

In the N-ERD vs. control group comparison, we found that all polymorphisms of *PPARG, IL10, TBXAS1, FANCC,* and *FCER1G* had a statistical association *p* < 0.04, OR < 1 in the codominant model, but with the dominant model the results had a similar tendency with the common allele in double doses *p* < 0.03, OR < 1 except *FCERIG-*rs7258588 that did not show a significative result. In the N-ERD vs. asthma comparison, *PPARG-rs2960421*, both SNPs of *IL10, PTGER2-rs1409165,* and *OBSCN-rs4465344* showed an association *p* < 0.04, OR < 1 in the codominant model, only *IL10-*rs1554286CC and *IL10-*rs1800872CC persisting with the effect in the dominant model (*p* = 0.006, OR = 0.51/*p* < 0.02, OR = 0.56). When comparing asthma vs. CG *TBXAS1-*rs13239058, *FANCC-*rs1326188 and *OBSCN-*rs465344 obtained *p* < 0.02 in the codominant model, *TBXAS1-*rs13239058CC had statistical significance in the dominant model *p* = 0.02, OR = 0.61, *FANCC*-rs132618AA *p* = 0.007, OR = 0.48, and *OBSCN*-rs465344GG *p* = 0.004, OR = 2.16 (see Table 4).

### 3.2. Results of the Second Stage

#### 3.2.1. Demographic and Clinical Characteristics

In the second stage, N-ERD were older than asthma and CG (*p* < 0.005), as equal as the first stage, there were more women in all groups mainly in patients, counts of blood eosinophils were higher in N-ERD vs. asthma (*p* < 0.001) and control group subjects (*p* = 0.03) in the same way in the asthma vs. CG comparison (*p* = 0.001). IgE in the asthma group had the highest titles in comparison with N-ERD and CG (*p* < 0.001), and allergy sensitization was distributed in a greater proportion in the asthma group (*p* < 0.001). Control group subjects showed better FEV_1_ values than patients (*p* < 0.001), between them there was no difference (see Table 5).

#### 3.2.2. Genetic Models in Replicative Phase

In the second stage, only *IL10*-rs1800872CC had an association in the comparison N-ERD vs. GC, *p* = 0.004, OR = 0.44, CI95% (0.25–0.78) with the dominant model but not in codominant or allelic models; no other polymorphism showed an association in any of the three comparisons (Table 6).

Assembling the results of the two stages, we identified that this SNP was associated with the codominant model and allelic model in N-ERD vs.CG *p* = 0.007, *p* = 0.005 in the same comparison, as the dominant model retained the statistical association *p* < 0.001, OR = 0.51, CI95% (0.35–0.73). In the comparison N-ERD vs. asthma, we showed an association only in the dominant model with genotype CC *p* = 0.009 OR = 0.61 CI95% (0.42–0.88).

Regarding *IL10*-rs1554286CC in N-ERD vs. CG, it had a statistical association in the codominant model *p* = 0.01, OR < 1, dominant *p* = 0.04, OR = 0.62 CI95% (0.43–0.89) and allelic *p* = 0.01, OR = 0.73 CI95% (0.57–0.89). When comparing N-ERD vs. asthma, we obtained the same magnitude in three models, and no other SNP showed an association with this strategy (Table S2).

#### 3.2.3. Clinical-Genotype Association

We undertook a stratified analysis in N-ERD patients of CC genotype-based in the dominant model (CC vs. CA + AA) with clinical variables, and this analysis showed that the women carrier of genotype CC were in the minor proportion in comparison CA + AA (*p* = 0.02) and Ig-E levels (*p* = 0.03) (Table S3). Logistic regression analysis had statistical significance for these variables *p* = 0.031/Exp(β) of 2.68 and 0.023/Exp(β) of 1.002 by each one.

## 4. Discussion

Based on our GoldenGate study of genetic susceptibility in Mexican-mestizo patients with N-ERD, we replicated 311 SNP into 53 candidate genomic regions spanning over 19 chromosomes associated with N-ERD, analyzed under the context of low risk (protection). The present study identified the association of *IL10-*rs1800872 with N-ERD in the Mexican-mestizo population.

Our group decided to develop a multistage genetic association study. Stage 1 analyzed the full set of SNPs genotyped in a fraction of samples, and a liberal *p*-value threshold was used to identify a subset of SNPs with putative associations. In the second stage, the SNPs identified from the first stage were retested in populations that were larger or of a similar size. The results of this can then be used to distinguish the few true-positive associations identified in stage 1 from the possible false-positive results that occur by chance. This kind of study can reduce the amount of genotyping required, without sacrificing power [17].

The *IL10* gene codes for the IL-10 protein, this gene is highly polymorphic [18], and many SNPs in this gene have been associated with allergic disease [19], wheezing in children [20], and childhood asthma phenotypes [21]. There is a metanalysis whose main objective was the association of SNPs of *IL10* with asthma, among which the analyzed polymorphisms rs1800872 (-592C/A) was included; Xue-yan Zheng showed the association of SNP with genotypes AC or AA to risk in Asian population and atopic asthma, but did not find this in Caucasians [22].

In our study, we detected an association with the common allele (C) of rs1800872, with the allelic and dominant model in the first stage and replicated the finding (low risk) with this last model in the second stage; in this sense, Xue-yan Zheng previously showed a haplotype, integrated by the common variants of three SNPs localized in promoter: rs1800896 (-1082G/A), rs1800871 (-819C/T), and rs1800872 (-592C/A), that included allele C of SNP of interest in our study, was associated with reduced risk of asthma [22]. Similar results found Holster when associated to genotype CC (wild) of rs1800871 and rs1800872 with less allergic rhinitis than the other variant genotypes [19]. This is the first-time that rs1800872 is associated with this sense in N-ERD.

It is probable that our finding is due to the distribution of the genotypes of this SNP in the Mexican population; Vargas-Alarcón has shown that frequencies of rs1800872 are different in comparison with other populations i.e., Caucasians [23], one of the two main populational contributions in the genome composition of the Mexican-mestizo [24], increasing the frequency almost twice as much in Caucasians and being 10 times higher than in Asian populations [25].

A study by Posada-Sanchez [26] describe frequencies similar to rs1800872 to Mexican residents in Los Angeles (USA) reported in HapMap [25], however, our results for this SNP in the CG are very similar to those reported by Martinez-Campos [27], although all studies agree that the C allele and their corresponding homozygous are the most frequent.

This phenomenon has been described in N-ERD genetics studies. There is evidence and documentations in other SNPs involved in mechanisms inherent to theoretical physiopathology as *LTC4* (-444A/C) is associated in Polish populations but not in Spanish and Caucasian-Americans from the USA, or HLA-DPB1 rs1042151 polymorphism to be a putative genetic factor in Korean N-ERD patients. Nevertheless, there is a lack of genetic association studies for this clinical entity in Spanish populations [28].

The ancestral component of our patients is like the report of the genomic diversity of Mexican-mestizo [24], and according to the two main groups of reference that integrate it [29]. The value obtained by Fst among the different study groups concluded that all participants were Mexican by ancestry, therefore we assume that the results of our genetic association are not conditioned by population stratification, for this reason, our subjects of study in the second stage were enrolled only by the antecedent of Mexican ancestry of at least two generations.

Regarding N-ERD, there is scarce evidence of *IL10* SNPs associated with N-ERD, i.e., Joo-Hee Kim analyzed SNPs with their response to inhalation rechallenge with lysine-aspirin after at least 1 year of regular treatment with antiasthmatic medications, however, there was no associated result with *IL10-*rs1800896 [30]. Moreover, S.-H. Kim showed the association of SNP in *IL10* associated with patients with aspirin-intolerant asthma and rhinosinusitis with *IL10-*rs1800896 but not rs1800872. In addition, the haplotype, including minor alleles in a Korean population, showed a risk with these clinical conditions [31].

IL-10 is a potent anti-inflammatory cytokine that protects the host from excessive tissue damage during the host’s defense against pathogens and has a pivotal role in the development and maintenance of immune tolerance and homeostasis [32], and plays a critical role in eosinophilic airway inflammation control [33], downregulating IL-6 and TNF-α by upper airway dendritic cells [34]. In the context of N-ERD, Stevens showed that IL-10 is decreased in nasal polyps from patients with this disease in comparison to patients with CRSwNP [35], on another hand in an epigenetic study Cheong reported that *IL10* gene is susceptible to hypomethylation [36].

The specific case of rs1800872 (-592C/A) suggests that IL-10 expression is regulated by the binding of Sp1 and Sp3 transcription factors to the upstream region of this polymorphism. The C to A change decreased the inhibitory effect of Sp1/Sp3 complex, favoring IL-10 expression in monocyte, B and T human cell lines [37]. The presence of the C allele is associated with high levels of IL-10 [38]; there are studies that have showed decreased levels of IL-10 in serum and sputum in asthma patients in comparison with healthy subjects [39,40].

We did not consider the association of *IL10*-rs1554286 because it did not show an evident association in the second stage.

The genetic association studies in N-ERD has opened many research fronts, and are grouped into six categories in a didactic way [41], which have contributed to reinforcing knowledge in this disease. However, many results have not been replicated due to small sample sizes or ethnic differences between study populations [42]. In this replicative study, our results of the first stage, 13 SNPs in eight gene regions had a positive association with N-ERD; *OBSCN-*rs465344 was discarded because it was not in a comparison associated with the disease of interest. *FCER1G-*rs7258588, *TBXAS1*-rs10487667, and *PTGER2-*rs1409165 did not show association in the dominant model. In the case of *PPARG-*rs2960421 and *FANCC-*rs1326188 no minor alleles were detected in the N-ERD group. In a similar case with *TBXAS1*-rs13239058 regarding *PPARG*, we decided validated rs1875796 because is a tag of 12 SNPs into the gene, contrary to rs413525 which is only a marker of itself. The SNPs of *IL10* had enough re-analysis criteria, and these alleles showed low risk in haplotype analysis and are in HWE, in addition to being the only ones who had an association in two comparisons according to the disease of interest by genetics models. It is likely that our method for selecting the SNPs in the second stage was very strict, but we considered increasing the sample size based on their minor allele frequency for evaluate the association in Mexican patients with N-ERD.

Concerning the clinical characteristics of our patients with N-ERD, they are very similar to other reports, and prevailed in women in the fourth decade of life, with high counts of eosinophils and around 50% had any type of allergy sensitivity [43,44]. In the first stage, we enrolled N-ERD patients with hypersensitivity to N-SAID though lysin-aspirin challenge, but in the second we invited patients with the antecedent of severe reaction (hospitalization or intubation) to N-SAID, documented in at least two episodes in medical records [45].

N-ERD is a very low prevalence asthma phenotype [2], and this phenomenon conditioned our sample size, being the reason for using a sample at convenience trying to adjust to other reports previously published (120 in the first stage and 100 in the second), with a range of 95–188 [12,46,47,48,49,50,51,52]. Nonetheless, we had power of 79.7 with the main genetic finding.

Unfortunately, we could not identify any clinical-genetic association when comparing the clinical characteristics by the dominant model in the N-ERD group, except for female gender and IgE levels; regarding the first characteristic, it was previously mentioned that N-ERD is more frequent in women. In the case of IgE, the values are considered normal according to the manufacturer’s specifications. Regarding the age, it is important to clarify that the N-ERD has a particular natural history of presentation, beginning in adulthood, on average at the age of 30 years. Rhinorrhea and nasal congestion are usually the first symptoms, subsequently complicated by nasal polyposis. Asthma and aspirin hypersensitivity develop 2–15 years later [53]; likewise, our patient population was enrolled by clinical characteristics and was not homogenized by therapeutic regimen [54]. There is evidence that the regimens of prescription according to EPOS [55], GINA [56] guidelines, and a N-ERD position paper with nasal and inhaled corticosteroids, decreased counts of eosinophils in nasal mucus, blood, and sputum, and increases FEV_1_ values, improving the quality life, reducing the underlying eosinophilic mucosal inflammation of the respiratory tract [1], modifying the biomarkers and clinical features characteristic of the disease. This involved to clinical-genetic associations but no impact in the genome of the patients.

Our study has some limitations, maybe the most important is that we enrolled patients according to the presence of clinical characteristics regardless of the type of treatment they were taking and the severity of the disease at the time of blood collection. These factors can modify the values of tests of respiratory function or biomarkers of N-ERD.

For the future, it will be interesting to analyze our findings together with the other polymorphisms in *IL10* as rs1800896 and/or rs1800871 located in the promoter region, as well as its serum and/or nasal IL-10 levels in a group of subjects without treatment, trying to understand the role of inflammatory mechanisms of *IL10*/IL-10 in N-ERD.

## 5. Conclusions

The Mexican patients with N-ERD have a clinical phenotype similar to other populations and the genetic ancestry of the Mexican-mestizo population did not differ. The most SNPs in genes associated with N-ERD described in other populations are not associated in the Mexican population; using this strategy of analysis only IL10-rs1800872 is associated to a low risk.

## Figures and Tables

**Table 1:** Demographic and clinical characteristics of the first stage.

Variable	N-ERD (N)	Asthma (A)	Control Group (CG)	*p* Value		
				N vs. CG	N vs. A	A vs. CG
N	120	180	180			
Age	43 (34–50)	39 (27–52)	27 (22–35)	<0.001	NS	<0.001
Female (%)	77 (64.1)	126 (70)	107 (59.4)	NS	NS	(0.04)
Eosinophils cell/mm^3^	400 (300–700)	170 (40–492)	100 (61–191)	<0.001	<0.001	0.006
IgE UI/L	125 (62–235)	229 (118–297)	88 (38–135)	0.001	0.001	<0.001
SPT+ n (%)	58 (48.33)	99 (55)	93 (51.66)	NS	NS	NS
FEV_1_ %	89 (73–106)	90 (80–101)	96 (87–106)	0.002	NS	0.001
Reversibility test	7 (2–13)	15 (8–21)	5 (0–5)	<0.001	<0.001	<0.001
TNF % of change in Nasal Provocation Test	54 (48–59)	10 (3–10)	3.6 (−5–5)	<0.001	<0.001 *	0.001 *

FEV_1_: forced expiratory volume in the first second. IgE: immunoglobulin E in International Units by litter. N-ERD: non-steroidal anti-inflammatory drugs exacerbated respiratory disease. NS: not significant. SPT+: positive skin prick test. TNF: total nasal flow. * Despite the statistical difference, the value of the change does not exceed the rhinomanometric criterion to be considered as positive (TNF >40%). Results expressed in medians and interquartile range.

**Table 2:** Ancestry.

Group	Ancestral Contribution		Asthma	N-ERD	CEU ^¥^	AME ^¥^
	CEU	AME				
Control Group	0.41	0.58	0.59	0.23	0.005	0.005
Asthma	0.44	0.56		0.47	0.005	0.005
N-ERD	0.48	0.52			0.005	0.005

Ancestral contribution (AC) of each study group with respect to the two reference populations that integrated the ancestry of Mexican-mestizo. ^¥^ Reference population, N-ERD: non-steroidal anti-inflammatory drugs exacerbated respiratory disease. AME: Amerindian. CEU: Caucasian European of Utah. Light shading shows *p* values among the study groups and dark shading shows *p* values among the AC study groups vs. reference population.

**Table 3:** Allelic frequencies, first stage.

*Gene*	SNP	Allele	N-ERD	GC	Asthma	N-ERD vs. CG			N-ERD vs. Asthma			Asthma vs. CG		
						*p* Value	OR	CI95%	*p* Value	OR	CI95%	*p* Value	OR	CI95%
***PPARG***	rs2960421	G	0.117	0.214	0.176	0.002	0.487	0.30–0.77	0.048	0.618	0.38–0.99	0.2057	0.7868	0.54–1.14
***IL10***	rs1554286	T	0.325	0.444	0.435	0.004	0.603	0.42–0.84	0.007	0.624	0.44–0.87	0.8208	0.9664	0.71–1.29
***TBXAS1***	rs13239058	T	0.146	0.232	0.162	0.010	0.566	0.36–0.87	0.593	0.883	0.56–1.39	0.019	0.641	0.44–0.93
***PPARG***	rs4135275	G	0.213	0.307	0.2725	0.010	0.608	0.41–0.89	0.09651	0.7205	0.48–1.06	0.3056	0.8444	0.61–1016
***IL10***	rs1800872	A	0.363	0.461	0.452	0.018	0.666	0.47–0.93	0.029	0.689	0.49–0.96	0.8263	0.9675	0.72–1.3
***PPARG***	rs1875796	T	0.425	0.517	0.4605	0.028	0.691	0.49–0.96	0.3937	0.8661	0.62–1.20	0.1328	0.7977	0.59–1.07
***TBXAS1***	rs10487667	G	0.313	0.399	0.3883	0.032	0.685	0.48–0.96	0.05815	0.7162	0.50–1.01	0.7717	0.9565	0.70–1.29
***FANCC***	rs1326188	C	0.075	0.129	0.073	0.038	0.550	0.31–0.97	0.9283	1.029	0.55–1.92	0.014	0.534	0.32–0.88
***FCER1G***	rs7528588	G	0.213	0.285	0.2331	0.047	0.677	0.46–0.99	0.5538	0.8876	0.59–1.31	0.1144	0.7631	0.54–1.06
***TBXAS1***	rs6962291	A	0.388	0.469	0.4635	0.050	0.717	0.51–0.99	0.06634	0.7323	0.52–1.02	0.8844	0.9784	0.72–1.31
***PTGER2***	rs1409165	C	0.108	0.129	0.169	0.458	0.824	0.49–1.37	0.040	0.599	0.36–0.98	0.1324	1.375	0.90–2.08
***OBSCN***	rs4653544	A	0.121	0.179	0.182	0.055	0.631	0.39–1.01	0.046	0.620	0.38–0.99	0.9225	1.019	0.64–1.49
***PTGER3***	rs1254600	T	0.367	0.430	0.344	0.121	0.767	0.54–1.07	0.5624	1.106	0.78–1.55	0.017	0.693	0.51–0.93
***TBXAS1***	rs2072190	C	0.471	0.458	0.344	0.760	1.053	0.75–1.46	0.001803	1.7	1.21–2.37	0.002	0.619	0.45–0.83
***TBXAS1***	rs2269997	C	0.400	0.503	0.401	0.899	0.170	0.64–1.24	0.002096	1.678	1.20–2.33	0.006	0.662	0.49–0.89
***CYSLTR2***	rs912278	C	0.363	0.413	0.327	0.212	0.807	0.57–1.13	0.3669	1.171	0.83–1.65	0.016	0.689	0.50–0.93
***TBXAS1***	rs17161326	A	0.288	0.346	0.261	0.137	0.764	0.53–1.09	0.4797	1.141	0.79–1.64	0.014	0.670	0.48–0.92

*CYSLTR2*: cysteinyl leukotriene receptor 2. *FANCC*: FA complementation group C. *FCER1G*: Fc fragment of IgE receptor Ig. *IL10*: interleukin 10. *OBSCN:* obscurin, cytoskeletal calmodulin, and titin-interacting RHoGEF. *PPARG*: peroxisome proliferator-activated receptor gamma. *PTGER2*: prostaglandin E receptor 2. *PTGER3*: prostaglandin E receptor 3. *TBXAS1*: thromboxane A synthase 1. OR: odds ratio.

**Table 4:** Genotype frequencies of single nucleotide polymorphisms (SNPs) associated with the first stage analysis.

Gene-rsID	N-ERD		Asthma		CG		N-ERD vs. CG			N-ERD vs. Asthma			Asthma vs. CG		
*PPARG*-rs2960421	*n*	%	*n*	%	*n*	%	*p* Value	OR	CI95%	*p* Value	OR	CI95%	*p* Value	OR	CI95%
**AA**	92	76.67	122	68.16	109	60.89	0.001	1		0.04	1		0.21	1	
**AG**	28	23.33	51	28.49	64	35.75		0.51	0.30–0.87		0.72	0.42–1.24		0.71	0.45–1.11
**GG**	0	0	6	3.352	6	3.352								0.89	0.27–2.85
**AA**	92	76.67	122	68.16	109	60.89	0.04	0.47	0.28–0.79	0.1	0.65	0.38–1.10	0.15	0.72	0.47–1.12
**AG + GG**	28	23.33	57	31.84	70	39.11									
***PPARG*-rs1875796**															
**CC**	39	32.5	53	29.61	37	20.67	0.02	1		0.42	1		0.1	1	
**CT**	60	50	88	49.16	99	55.31		0.57	0.33–0.99		0.92	0.54–1.57		0.62	0.37–1.03
**TT**	21	17.5	38	21.23	43	24.02		0.46	0.23–0.92		0.75	0.38–1.47		0.61	0.33–1.13
**CC**	39	32.5	53	29.61	37	20.67	0.02	0.54	0.31–0.91	0.59	0.87	0.53–1.43	0.05	0.61	0.38–1.00
**CT + TT**	81	67.5	126	70.39	142	79.33									
***PPARG-*rs4135275**															
**AA**	74	61.67	94	52.51	84	46.93	0.009	1		0.1	1		0.27	1	
**AG**	41	34.17	73	40.78	80	44.69		0.58	0.35–0.94		0.71	0.43–1.16		0.81	0.52–1.25
**GG**	5	4.167	12	6.704	15	8.38		0.37	0.13–1.09		0.52	0.17–1.56		0.71	0.31–1.61
**AA**	74	61.67	94	52.51	84	46.93	0.01	0.54	0.34–0.88	0.11	0.68	0.42–1.10	0.29	0.79	0.51–1.21
**AG + GG**	46	38.33	85	47.49	95	53.07									
***TBXAS1*-rs13239058**															
**CC**	88	73.33	126	70.39	106	59.22	0.01	1		0.59	1		0.02	1	
**CT**	29	24.17	48	26.82	63	35.2		0.55	0.32–0.93		0.86	0.50–1.47		0.64	0.40–1.01
**TT**	3	2.5	5	2.793	10	5.587		0.36	0.09–1.35		0.85	0.20–3.68		0.42	0.13–1.26
**CC**	88	73.33	126	70.39	106	59.22	0.01	0.52	0.31–0.87	0.58	0.86	0.51–1.44	0.02	0.61	0.39–0.94
**CT + TT**	32	26.67	53	29.61	73	40.78									
***TBXAS1*-rs10487667**															
**TT**	54	45	66	36.87	62	34.64	0.02	1		0.05	1		0.75	1	
**GT**	57	47.5	87	48.6	91	50.84		0.71	0.43–1.17		0.8	0.49–1.30		0.89	0.57–1.41
**GG**	9	7.5	26	14.53	26	14.53		0.39	0.17–0.92		0.42	0.18–0.97		0.93	0.49–1.79
**TT**	54	45	66	36.87	62	34.64	0.07	0.64	0.40–1.03	0.15	0.71	0.44–1.14	0.65	0.9	0.58–1.39
**GT + GG**	66	55	113	63.13	117	65.36									
***TBXAS1*-rs6962291**															
**TT**	48	40	57	31.84	49	27.37	0.05	1		0.08	1		0.94	1	
**AT**	51	42.5	78	43.58	93	51.96		0.55	0.33–0.94		0.77	0.46–1.30		0.72	0.44–1.17
**AA**	21	17.5	44	24.58	37	20.67		0.57	0.29–1.12		0.56	0.29–1.08		1.02	0.57–1.82
**TT**	48	40	57	31.84	49	27.37	0.02	0.56	0.34–0.92	0.14	0.7	0.43–1.13	0.35	0.8	0.51–1.27
**AT + AA**	72	60	122	68.16	130	72.63									
***IL10*-** **rs1554286**															
**CC**	57	47.5	57	31.84	55	30.73	0.004	1		0.007	1		0.82	1	
**CT**	48	40	88	49.16	89	49.72		0.52	0.31–0.86		0.54	0.32–0.90		0.95	0.59–1.53
**TT**	15	12.5	34	18.99	35	19.55		0.41	0.20–0.84		0.44	0.21–0.89		0.93	0.51–1.70
**CC**	57	47.5	57	31.84	55	30.73	0.003	0.49	0.30–0.79	0.006	0.51	0.32–0.83	0.81	0.94	0.60–1.48
**CT + TT**	63	52.5	122	68.16	124	69.27									
***IL10*-** **rs1800872**															
**CC**	51	42.5	54	30.17	53	29.61	0.02	1		0.03	1		0.88	1	
**CA**	51	42.5	88	49.16	88	49.16		0.6	0.35–1.00		0.61	0.36–1.02		0.98	0.60–1.58
**AA**	18	15	37	20.67	38	21.23		0.49	0.24–0.97		0.51	0.26–1.01		0.95	0.52–1.72
**CC**	51	42.5	54	30.17	53	29.61	0.02	0.56	0.35–0.92	0.02	0.56	0.35–0.93	0.9	1.02	0.65–1.61
**CA + AA**	69	57.5	125	69.83	126	70.39									
***FANCC*-rs1326188**															
**AA**	102	85	154	86.03	134	74.86	0.03	1		0.91	1		0.01	1	
**AC**	18	15	24	13.41	44	24.58		0.53	0.29–0.98		1.13	0.58–2.19		0.47	0.27–0.82
**CC**	0	0	1	0.559	1	0.559								0.87	0.05–1.404
**AA**	102	85	154	86.03	134	74.86	0.03	0.52	0.28–0.96	0.8	1.08	0.56–2.09	0.007	0.48	0.28–0.83
**AC + CC**	18	15	25	13.97	45	25.14									
***FCER1G*-rs7258588**															
**CC**	72	60	111	62.01	92	51.4	0.04	1		0.58	1		0.12	1	
**CG**	45	37.5	53	29.61	72	40.22		0.79	0.49–1.29		1.3	0.79–2.14		0.61	0.38–0.95
**GG**	3	2.5	15	8.38	15	8.38		0.25	0.07–0.91		0.3	0.08–1.10		0.82	0.38–1.78
**CC**	72	60	111	62.01	92	51.4	0.14	0.7	0.44–1.12	0.72	1.08	0.67–1.74	0.04	0.64	0.42–0.98
**CG + GG**	48	40	68	37.99	87	49.72									
***PTGER2*- rs1409165**															
**TT**	94	78.33	122	68.16	137	76.54	0.45	1		0.03	1		0.13	1	
**TC**	26	21.67	54	30.17	38	21.23		0.99	0.56–1.75		0.62	0.36–1.07		1.59	0.98–2.58
**CC**	0	0	3	1.676	4	2.235								0.84	0.18–3.83
**TT**	94	78.33	122	68.16	137	76.54	0.71	0.9	0.51–1.57	0.05	0.59	0.34–1.01	0.07	1.52	0.95–2.43
**TC + CC**	26	21.67	57	31.84	42	23.46									
***OBSCN*-rs465344**															
**GG**	93	77.5	119	81.5	120	67	0.43	1		0.04	1		0.003	1	
**GA**	25	20.8	25	17.1	53	29.6		1.27	0.69–2.37		0.60	0.35–1.05		0.47	0.27–0.81
**AA**	2	1.6	2	1.3	6	3.3		1.27	0.17–9.25		0.43	0.08–2.18		0.33	0.06–1.69
**GG**	93	77.5	119	81.5	120	67	0.51	1.27	0.70–2.32	0.06	0.59	0.34–1.03	0.004	2.16	1.28–3.64
**GA + AA**	27	22.5	27	18.4	59	32.9									

CG: control group. *FANCC*: FA complementation group C. *FCER1G*: Fc fragment of IgE receptor Ig. *IL10*: interleukin 10. N-ERD: non-steroidal anti-inflammatory drugs exacerbated respiratory disease. *PPARG*: peroxisome proliferator-activated receptor gamma. *PTGER2*: prostaglandin E receptor 2. *TBXAS1*: thromboxane A synthase 1. *OBSCN*: obscurin, cytoskeletal calmodulin, and titin-interacting RhoGEF. OR: odds ratio.

**Table 5:** Demographic and clinical characteristics of the included subjects in the second stage.

Variable	N-ERD (N)	Asthma (A)	Control Group (CG)	*p* Value		
				N vs. CG	N vs. A	A vs. CG
N	100	96	116			
Age	42 (33–53)	36 (27–46)	34 (26–43)	0.001	0.005	0.09
Female n (%)	70 (70)	66 (68.75)	64 (55.17)	0.02	0.84	0.04
Eosinophils cell/mm^3^	400 (215–700)	300 (200–428)	136 (84–219)	0.001	0.03	0.001
IgE UI/L	107 (48–254)	266 (131–500)	62 (18–107)	0.001	0.001	0.001
SPT+ n (%)	30 (30)	82 (85.41)	51 (43.96)	0.03	0.001	0.001
FEV_1_ %	86 (73–97)	90 (76–99)	96 (89–109)	0.001	0.11	0.001
Reversibility Test	10 (5–13)	12 (6–16)	3 (0–5)	0.001	0.10	0.001

FEV_1_: forced expiratory volume in the first second. IgE: immunoglobulin E in International Units by litter. N-ER: non-steroidal anti-inflammatory drugs exacerbated respiratory disease. SPT+: positive skin prick test. Results expressed in medians and interquartile range.

**Table 6:** Genotype and allelic frequencies of the second stage.

Gene-rsID	N-ERD *n* = 100		Asthma *n* = 96		Control *n* = 116		N-ERD vs. CG			N-ERD vs. Asthma			Asthma vs. CG		
							*p*	OR	CI95	*p*	OR	CI95	*p*	OR	CI95
***PPARG-rs1875796***	***n***	**%**	***n***	**%**	***n***	**%**									
CC	32	32	31	32.29	41	35.34	0.41	1		0.8	1		0.6	1	
CT	52	52	47	48.96	43	37.07		1.5	0.83–2.86		1.1	0.56–2.01		1.4	0.77–2.69
TT	16	16	18	18.75	32	27.59		0.6	0.30–1.36		0.9	0.37–1.98		0.7	0.35–1.56
CC	32	32	31	32.29	41	35.34	0.60	1.2	0.65–2.04	0.96	1	0.55–1.84	0.6	1.1	0.64–2.03
CT + TT	68	68	65	67.71	75	64.66									
C	116	58	109	56.77	125	53.88	0.38	0.8	0.57–1.23	0.80	1	0.63–1.41	0.6	0.9	0.60–1.30
T	84	42	83	43.23	107	46.12									
***IL10*-rs1554286**															
CC	35	35	30	31.25	36	31.03	0.68	1		0.72	1		1	1	
CT	46	46	48	50	58	50		0.81	0.44–1.49		0.8	0.43–1.54		1	0.53–1.84
TT	19	19	18	18.75	22	18.97		0.9	0.41–1.91		0.90	0.40–2.03		1	0.44–2.16
CC	35	35	30	31.25	36	31.03	0.53	0.8	0.47–1.47	0.89	1	0.52–1.75	0.6	0.9	0.48–1.57
CT + TT	65	65	58	60.42	80	68.97									
C	116	58	108	56.25	130	56.03	0.68	0.9	0.62–1.35	0.72	0.9	0.62–1.38	1	1	0.67–1.45
T	84	42	84	43.75	102	43.96									
***IL10-rs1800872***															
CC	47	47	37	38.54	33	28.45	0.14	1		0.92	1		0.1	1	
CA	29	29	45	46.88	59	50.86		0.3	0.18–0.64		0.50	0.26–0.95		0.7	0.37–1.25
AA	24	24	14	14.58	24	20.69		0.70	0.34–1.44		1.3	0.61–2.96		0.5	0.23–1.16
CC	47	47	37	38.54	33	28.45	0.004	0.4	0.25–0.78	0.23	0.70	0.40–1.24	0.1	0.6	0.35–1.12
CA + AA	53	53	59	61.46	83	71.55									
C	123	61.5	119	61.98	125	53.88	0.11	0.7	0.49–1.07	0.92	1	0.67–1.53	0.1	0.7	0.48–1.05
A	77	38.5	73	38.02	107	46.12									

CG: control group. *IL10:* interleukin 10. *PPARG*: peroxisome proliferator-activated receptor gamma. N-ERD: non-steroidal anti-inflammatory drugs exacerbated respiratory disease. OR: odds ratio.

## Supplementary Materials

The following are available online at https://www.mdpi.com/2218-273X/10/1/104/s1, Figure S1: Haplotype analysis of TBXAS1 associated with N-ERD, Table S1: Haplotype frequency of *IL10* (rs155286 and rs1800872), Table S2: Genotype frequencies (first and second stages), Table S3: Clinical association by the dominant model in N-ERD patients.
