# Supplementary material for: IL10 rs1800872 Is Associated with Non-Steroidal Anti-Inflammatory Drugs Exacerbated Respiratory Disease in Mexican-Mestizo Patients
Source: Biomolecules. 2020 Jan 7;10(1):104. doi: 10.3390/biom10010104 (PMC7023146; doi:10.3390/biom10010104)
Supplement: Supplementary file 1 [file biomolecules-10-00104-s001.pdf]

Supplementary material.

Gene	N-ERD vs. CG	N-ERD vs. Asthma	Asthma vs. CG
TBAXS1	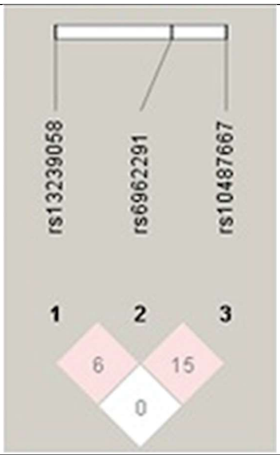	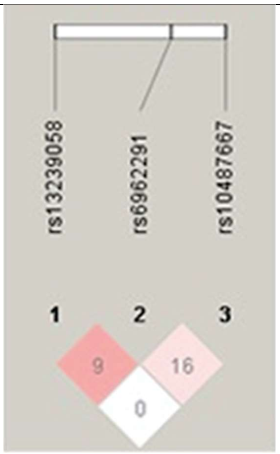	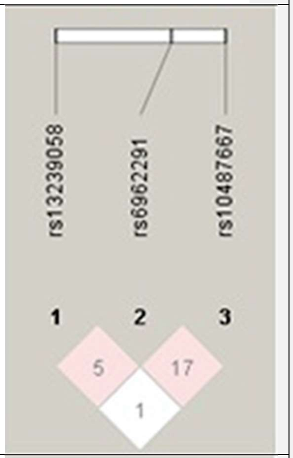
PPARG	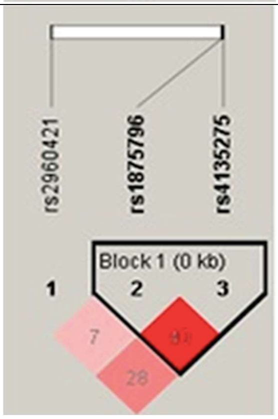	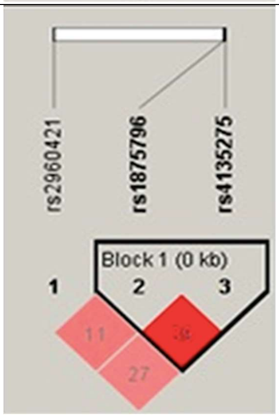	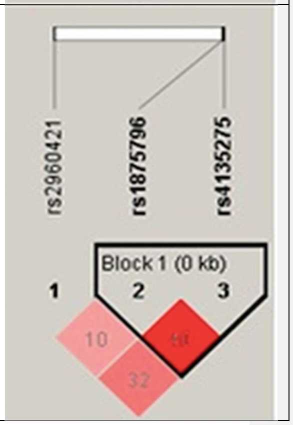

Figure S1. Haplotype analysis of TBXAS1 associated with N-ERD. N-ERD: non-steroidal anti-inflammatory drugs exacerbated respiratory disease. CG: control group. *PPARG*: peroxisome proliferator-activated receptor gamma. *TBXAS1*: thromboxane A synthase 1.

Table S1. Haplotype frequency of *IL10* (rs155286 and rs1800872)

		**N-ERD**		**CG**		**Asthma**		**N-ERD vs.CG**			**N-ERD vs. Asthma**			**Asthma vs. CG**		
**Haplotype**	**F**	***n***	**%**	***n***	**%**	***n***	**%**	***p***	**OR**	**CI95%**	***p***	**OR**	**CI95%**	***p***	**OR**	**CI95%**
**CC**	0.578	153	0.64	189	0.54	194	0.55	**0.015**	0.65	0.47–0.92	**0.04**	0.69	0.49–0.97	0.71	0.94	0.70–1.27
**TA**	0.397	78	0.33	157	0.45	152	0.43	**0.003**	1.67	1.18–2.35	**0.01**	1.57	1.12–2.22	0.7	1.05	0.78–1.42
**CA**	0.025	9	0.04	6	0.02	6	0.02	0.12	0.44	0.15–1.26	0.12	0.44	0.15–1.26	1	1	0.31–3.13

CG: control group. F: frequency. N-ERD: non-steroidal anti-inflammatory drugs exacerbated respiratory disease. OR: odds ratio.

Table S2. Genotype frequencies (first and second stages).

	N-ERD						Asthma						Control Group (CG)						N-ERD vs. CG			N-ERD vs. Asthma			Asthma vs. CG		
**Gene-rsID**	**120/240**		**100/200**		**220/440**		**179 /358**		**96 /192**		**275/550**		**179 /358**		**116 /232**		**295 /590**		***p***	**OR**	**CI95**	***p***	**OR**	**CI95**	***p***	**OR**	**CI95**
PPARG-rs1875796	**1**	**1%**	**2**	**2%**	**t**	**t%**	**1**	**1%**	**2**	**2%**	**t**	**t%**	**1**	**1%**	**2**	**2%**	**t**	**t%**									
CC	39	32.5	32	32	71	32.3	53	29.61	31	32.29	84	30.55	37	20.67	41	35.34	78	26.44	0.02	1		0.4	1		0.12	1	
CT	60	50	52	52	112	50.9	88	49.16	47	48.96	135	49.09	99	55.31	43	37.07	142	48.14		0.86	0.57–1.30		0.98	0.65–1.46		0.88	0.59–1.30
TT	21	17.5	16	16	37	16.8	38	21.23	18	18.75	56	20.36	43	24.02	32	27.59	75	25.42		0.54	0.32–0.90		0.78	0.46–1.31		0.69	0.43–1.10
CC	39	32.5	32	32	71	32.3	53	29.61	31	32.29	84	30.55	37	20.67	41	35.34	78	26.44	0.14	0.75	0.51–1.10	0.68	0.92	0.63–1.35	0.27	0.81	0.56–1.17
CT+TT	81	67.5	68	68	149	67.7	126	70.39	65	67.71	191	69.45	142	79.33	75	64.66	217	73.56									
C	138	57.5	116	58	254	57.7	194	54.2	109	56.77	303	55.09	173	48.3	125	53.88	298	50.51	0.02	0.74	0.58–0.95	0.4	0.89	0.69–1.15	0.12	0.83	0.65–1.05
T	102	42.5	84	42	186	42.3	164	45.8	83	43.23	247	44.91	185	51.7	107	46.12	292	49.49									
IL10-**rs1554286**																											
CC	57	47.5	35	35	92	41.8	57	31.84	30	31.25	87	31.64	55	30.73	36	31.03	91	30.85		1			1			1	
CT	48	40	46	46	94	42.7	88	49.16	48	50	136	49.45	89	49.72	58	50	147	49.83	0.01	0.63	0.42–0.93	0.03	0.65	0.44–0.96	0.83	0.96	0.66–1.40
TT	15	12.5	19	19	34	15.5	34	18.99	18	18.75	52	18.91	35	19.55	22	18.97	57	19.32		0.59	0.35–0.98		0.61	0.36–1.04		0.95	0.59–1.53
CC	57	47.5	35	35	92	41.8	57	31.84	30	31.25	87	31.64	55	30.73	36	31.03	91	30.85	0.04	0.62	0.43–0.89	0.01	0.64	0.44–0.93	0.83	0.96	0.67–1.37
CT+TT	63	52.5	65	65	128	58.2	122	68.16	58	60.42	188	68.36	124	69.27	80	68.97	204	69.15									
C	162	67.5	116	58	278	63.2	202	56.4	108	56.25	310	56.36	199	55.6	130	56.03	329	55.76	0.01	0.73	0.57–0.94	0.02	0.75	0.58–0.97	0.83	0.97	0.77–1.23
T	78	32.5	84	42	162	36.8	156	43.6	84	43.75	240	43.64	159	44.4	102	43.96	261	44.24									

IL10-rs1800872																											
CC	51	42.5	47	47	98	44.5	54	30.17	37	38.54	91	33.09	53	29.61	33	28.45	86	29.15		1			1			1	
CA	51	42.5	29	29	80	36.4	88	49.16	45	46.88	133	48.36	88	49.16	59	50.86	147	49.83	0.007	0.47	0.32–0.71	0.09	0.55	0.37–0.83	0.09	1.79	1.20–2.66
AA	18	15	24	24	42	19.1	37	20.67	14	14.58	51	18.55	38	21.23	24	20.69	62	21.02		0.59	0.36–0.96		0.76	0.46–1.25		1.3	0.79–2.15
CC	51	42.5	47	47	98	44.5	54	30.17	37	38.54	91	33.09	53	29.61	33	28.45	86	29.15	0.000 3	0.51	0.35–0.73	0.009	0.61	0.42–0.88	0.3	0.83	0.58–1.18
CA+AA	69	57.5	53	53	122	55.5	125	69.83	59	61.46	184	66.91	126	70.39	83	71.55	209	70.85									
C	153	63.8	123	61.5	276	62.7	196	54.7	119	61.98	315	57.27	194	54.2	125	53.88	319	54.07	0.005	0.69	0.54–0.90	0.08	0.79	0.61–1.02	0.27	0.87	0.69–1.10
A	87	36.3	77	38.5	164	37.3	162	45.3	73	38.02	235	42.73	164	45.8	107	46.12	271	45.93									

1(n of *first stage*, by each group), 1%, *(percentage of first stage*, by each group), 2(n of *second stage*, by each group), 2%, *(percentage of second stage*, by each group), t (sum of 1 and 2 stage, by each group), t% (percentage of n total, by each group), CG: control group. *IL10:* interleukin 10. N-ERD: non-steroidal anti-inflammatory drugs exacerbated respiratory disease. PPARG: peroxisome proliferator-activated receptor gamma.

Commented [MM1]: Please review use of underline and italics for all uses

Table S3. Clinical association by the dominant model in N-ERD patients.

Variable	CC	CA + AA	*p*-value
**N**	47	53	
**Age**	41 (29–54)	45 (35–51)	0.28
**Female n (%)**	27 (58)	42 (79)	0.02
**Eosinophils**	400 (300–650)	400 (210–700)	0.75
**SPT+ n (%)**	12 (26)	17 (32)	0.75
**IgE**	187 (52–371)	90 (40–160)	0.03
**FEV_1 %**	83 (78–91)	88 (79–99)	0.93

FEV_1: forced expiratory volume in the first-second post-bronchodilator. IgE: immunoglobulin E. SPT+: positive skin prick test. Results expressed in median and interquartile range.
